# Use of Vocational Rehabilitation Supports for Postsecondary Education Among Transition-Age Youth on the Autism Spectrum

**DOI:** 10.1007/s10803-019-03972-8

**Published:** 2019-03-08

**Authors:** Jessica E. Rast, Anne M. Roux, Paul T. Shattuck

**Affiliations:** grid.166341.7 0000 0001 2181 3113 A.J. Drexel Autism Institute, Drexel University, 3020 Market St., Suite 560, Philadelphia, PA 19130 USA

**Keywords:** Autism, Vocational rehabilitation, Postsecondary education, Employment, Services

## Abstract

Transition-age youth with autism (TAY-ASD) experience poor employment outcomes and gaps in services that could assist them in securing jobs. Vocational rehabilitation (VR) is a source of public assistance for people with disabilities seeking employment and TAY-ASD are a growing segment of VR service users. Postsecondary education (PSE) is essential for building vocational skills, contributing to employment satisfaction and better wages. VR provides services to support PSE success. Fewer TAY-ASD received PSE training from VR (18%) than TAY with other disabilities (32%), but more than TAY with an intellectual disability (15%). TAY-ASD who received PSE training were more likely to exit VR with a job. The importance of PSE to employment should be considered in TAY-ASD who seek employment supports.

## Introduction

Most of the estimated 50,000 youth with autism who turn 18 every year are capable of employment and have valuable contributions to make in the workforce and in their communities. Employment allows people to earn wages, increases independence, and improves quality of life (García-Villamisar et al. 2002; Hendricks 2010). It may also reduce reliance on government funds and public benefits. The rate of participation in employment is lower in adults with disabilities than in adults without them (Kraus et al. 2018).

A key barrier to establishing a career pathway is the lack of access to and completion of postsecondary education (PSE). The importance of PSE in job acquisition and career building has never been higher (Carnevale and Rose 2015; Carnevale et al. 2013). The Great Recession differentially impacted workers without PSE; those with some PSE were less likely to lose their job during the recession, and more jobs were added after the recession that were filled by workers with PSE (Carnevale et al. 2016). Adults with a high school diploma or less now have the highest unemployment rates and the lowest wages (Department of Labor 2018), while attainment of any type of PSE degree (Associate’s, Vocational-Technical, Bachelor’s, or graduate education) has been associated with career acquisition in young adults (Vuolo et al. 2014). Attainment of PSE is associated with enhanced economic mobility even in jobs where a postsecondary credential is specifically not required. Therefore, PSE is often a positive return on investment in terms of future earnings and career mobility (Baum et al. 2013; Haskins et al. 2009).

Enrollment in PSE for transition-age youth (TAY) with disabilities is lower than in the general population (Newman et al. 2011). Enrollment for TAY with autism (TAY-ASD) is lower than that of many students with other disabilities (Newman et al. 2011; Roux et al. 2015; Shattuck et al. 2012b). Fewer than half (44%) of TAY-ASD who received special education services attended some type of PSE in the 8 years following high school, compared to two-thirds of young adults in the general population (Newman et al. 2011).

The rate of participation in employment for TAY with disabilities and autism is correspondingly low (Newman et al. 2011). Fewer TAY-ASD have a job in the first years following high school than TAY in special education with other disabilities (Newman et al. 2011; Roux et al. 2015). About 40% never work between high school and their early 20s (Roux et al. 2015). Four to six years after exiting high school, 31% of TAY-ASD did not have a job or any PSE experience (Wei et al. 2014). Over one-fourth (28%) of those who did not work or continue their education between high school and their early 20s also had no services that could have supported their path to employment (Roux et al. 2015). Supporting opportunities for PSE among TAY-ASD could improve employment outcomes.

It is also important to recognize that many students with disabilities need accommodations and supports to be successful in PSE. However, the types and amounts of support available in most PSE settings are not as comprehensive as services they may have received through special education in secondary school. PSE settings are only required to offer reasonable accommodations that allow students to access academics. The types of supports available to students vary widely, and institutions are not required to provide supports that would alter the academic program or increase financial burden on the institution (U.S. Department of Education Office of Special Education and Rehabilitative Services 2017). Of students with autism who attended some type of PSE, 35% received accommodations and supports from their institution because of their disability (Newman et al. 2011). This was lower than students with sensory or physical disabilities including hearing (53%), visual (59%), and orthopedic impairments (55%). One-third of students with autism (34%) received help outside of what was provided by their PSE institution.

In addition to supports and accommodations through the PSE institution, another potential source of support is PSE training services through Vocational Rehabilitation (VR). VR funds employment services in the United States for people with disabilities including autism. VR services are administered by states using a combination of federal and state funds, and delivered through an extensive network of local VR offices and community rehabilitation providers. VR is tasked with helping individuals with disabilities “prepare for and engage in competitive integrated employment,” (34 CFR § 361.1). Approximately 50–60% of TAY-ASD are employed when they exit VR services (Kaya et al. 2016; Roux et al. 2018b). Factors known to influence VR outcomes for TAY-ASD include gender, receipt of public benefits including supplemental security income, race, state of residence, and level of education at entry (Kaya et al. 2016; Migliore et al. 2012). VR-provided job placement and on-the-job supports are associated with a higher likelihood of attaining employment among youth and adults with autism (Kaya et al. 2016; Lawer et al. 2009; Migliore et al. 2012; Schaller and Yang 2005; Sung et al. 2015).

If PSE is deemed necessary for achieving an eligible participant’s employment goals, VR can provide financial support to offset costs associated with PSE, including disability-related expenses such as educational support services or personal assistants (U.S. Department of Education Office of Special Education and Rehabilitative Services 2017). VR can also play a role in connecting individuals with the office at the PSE institution that provides disability services. The provision of PSE training through VR is lower in service users with intellectual or developmental disabilities (IDD) or psychiatric disabilities than for service users with sensory or physical disabilities (O’Neill et al. 2015). However, little is known about how often TAY-ASD access this service through VR. Among all VR service users, receipt of PSE training through VR is associated with higher wages at VR exit, an effect that is the strongest for TAY with IDD or psychiatric disabilities (O’Neill et al. 2015). However, the role of PSE training in employment outcomes of TAY-ASD is not well understood.

Supporting PSE is a growing priority in VR. Changes to VR required by the Workforce Innovation and Opportunity Act (WIOA) increase the emphasis on preparing students for work and clarifies the role of VR in PSE. One of six new performance measures for VR success is “advancement in degree status between application and closure.” WIOA also specifies that VR funding can be used for higher education costs as long as other sources of payment, such as grants, have been sought first (34 CFR § 361.48). Understanding more about the subpopulation of TAY-ASD who receive PSE services is important for assessing the success of WIOA changes.

This study aims to use VR administrative data to: (1) describe rates of PSE training services among TAY-ASD versus TAY with other IDDs, and all other TAY receiving VR services; (2) examine the characteristics of TAY-ASD who receive PSE training services compared to TAY-ASD who do not; and (3) estimate the effect of VR-funded PSE training services on subsequent exit from VR with employment for TAY-ASD.

Examining these aims will help illuminate how many TAY-ASD access PSE training services through VR, and how receipt of these services is associated with employment success at VR exit. As PSE is of increasing importance to employment and an expanding focus of VR legislation, understanding how TAY-ASD access PSE through VR is important. This study will be among the first to describe the use of PSE training services through VR for TAY-ASD and specifically consider the association of PSE training services and exit from VR with employment. Findings may help more TAY-ASD access PSE training services through VR by exploring their utility in successful employment.

## Methods

This study used data from the U.S. Department of Education’s Rehabilitation Service Administration Case Service Report (RSA-911) for federal fiscal year (FFY) 2015. The RSA-911 is an administrative record of all people who had a VR case closed in a given year. The RSA-911 includes information on demographics, disability characteristics, services received from VR, whether employment was attained, and other reasons for case closure. Analysis of this secondary dataset was deemed exempt by the Drexel University Institutional Review Board.

We examined TAY ages 14–24 at the time of application who received services from VR and had a primary or secondary cause of disability of autism (n = 12,073). In comparison, we looked at TAY ages 14–24 who received services from VR and had a primary or secondary cause of disability of intellectual disability, Cerebral Palsy, or traumatic brain injury, but not autism (n = 21,102). We also looked at all other TAY with disabilities who used VR services (n = 85,972). Primary and secondary causes of disability were considered in this study. Analyses were limited to those who lived in the 50 U.S. states or Washington D.C.

### Measures

The main outcome was employment at the time of exit from VR. The RSA defines employment as a full- or part-time job in an integrated setting (i.e., in which not all employees have disabilities), paying below, at, or above the minimum wage, or self-employment (Department of Education Office of Special Education and Rehabilitative Services 2014). Employment in a state agency-managed business enterprise program, working as an unpaid family worker, and working as a homemaker were not considered employment for this study.

PSE training through VR is defined by RSA as services to help the individual improve educationally or vocationally, including educational training for graduate college, 4-year college, junior college, or vocational training.

We used three categories to organize covariates: individual characteristics, VR characteristics, and receipt of other VR services. We selected variables that prior research has shown to be associated with better employment outcomes for adults with autism who use VR services. Individual characteristics included age, race, ethnicity, gender, receipt of SSDI or SSI at application, type of insurance at application, primary source of support at application, the highest level of education completed at the time of application, and secondary student status at the time of application. VR characteristics included being deemed to have a most significant disability, referral to VR from a primary or secondary school, and the mean number of days between signing of the individualized plan for employment and VR exit. Receipt of other VR services included job placement, on-the-job supports for supported employment, on-the-job supports short term, job search assistance, job readiness training, and VR counseling. Job placement services are defined by RSA as a referral to a specific job resulting in an interview, whether or not the individual obtained the job. On-the-job supports are defined as support services provided to an individual who has been placed in employment in order to stabilize the placement and enhance job retention; this may include short-term job coaching for persons who do not have a supported employment goal in their individualized plan for employment.

We also examined the advancement of PSE in TAY during VR. This measure captures TAY who received a degree from a PSE institution, attained a license or certificate, or attended some PSE but did not receive a degree or certificate. The goal of PSE training is advancement of PSE, but they do not necessarily occur together. This measure captures TAY who attempt to gain further PSE.

### Data Analysis

For Aim 1, we estimated proportions and means of the covariates and compared these between TAY-ASD, TAY with other IDDs, and all other TAY. We used logistic regression to compare the distribution of variables between the groups and reported significance at the p < 0.01 level because of the large number of comparisons made in the study. Advancement of PSE was examined as the percentage of TAY who exited VR with any education beyond high school completion. This included any PSE, technical certificate, or license attainment, with or without degree attainment. We excluded TAY who already had some PSE experience, as we would not be able to tell what experience was gained before or during VR service access. For Aim 2, we compared the distributions of all covariates between TAY-ASD who received PSE training services and TAY-ASD who did not receive PSE training services. All variables were missing fewer than 10% of observations. Most were missing fewer than 5% of observations, with only some VR service variables missing more than 5%.

For Aim 3, we used generalized boosted regression modeling (GBM) to estimate propensity scores in the *twang* package for Stata (Cefalu et al. 2015). In the context of this study, the propensity score is the predicted probability that a TAY-ASD would receive PSE training services, estimated from all other observed variables. Control variables can be seen in Table 1. Machine learning techniques, and particularly boosted regression, are less sensitive to propensity score model misspecification than other regression techniques (Lee et al. 2010) and are increasingly used to calculate propensity scores. Parametric models of estimation have assumptions that must be checked, such as variable distribution and specification of interaction, and if misspecified will lead to biased estimates. Nonparametric machine learning techniques do not have this same potential for bias. Propensity score weighting, as opposed to matching or stratification, includes the propensity score in the final outcome analysis and increases sensitivity to the misspecification of the propensity score model (Rubin 2004). This method also allows retention of more observations in our sample, where other methods, including propensity score matching, often lose cases that have no counterpart with a similar propensity score. Balance of the calculated propensity scores was assessed by calculating the standardized effect size (the mean in the treatment group minus the mean in the control group divided by the pooled sample standard deviation) and using a t-statistic or chi-squared statistic to test for significant difference between the groups after the weights were applied. Standardized difference was useful in this scenario as it is not influenced by sample size (Austin and Stuart 2015). To estimate the average treatment effect (ATE), the inverse probability treatment weight (IPTW) was calculated from the propensity score (p) [control cases weight = 1/p(x_i_); treatment weight = 1/(1 − p(x_i_))]. This weight was then applied as a probability weight using Stata svy commands and used in outcome analysis (Cefalu et al. 2015).

**Table 1:** Characteristics and service patterns of transition age youth with autism, IDD, and all other causes of disability

	Autism (n = 12,073)	Other IDD^a^ (n = 21,102)	All other TAY (n = 85,972)
	n (%)	n (%)	n (%)
Demographic characteristics			
Age (mean)	12,073 (19.0)	21,102 (19.3)***	85,927 (18.9)***
Gender, male	10,037 (83.1)	12,032 (57.0)***	49,591 (57.7)***
Race			
White	10,275 (85.1)	13,399 (63.5)***	62,759 (73.0)***
Black	1,186 (9.8)	6627 (31.4)***	18,619 (21.7)***
Other/multiple	612 (5.1)	1076 (5.1)***	4594 (5.3)***
Ethnicity—Hispanic or Latino	825 (6.8)	2191 (10.4)***	12,736 (14.8)***
Received SSDI at application	688 (5.7)	1529 (7.2)***	3039 (3.5)***
Received SSI at application	3497 (29.0)	8545 (40.5)***	12,387 (14.4)***
Type of insurance at time of application			
Private	5191 (43.0)	4318 (20.5)***	28,419 (33.1)***
Public	3751 (31.1)	10,607 (50.3)***	28,362 (33.0)***
Both private and public	658 (5.5)	911 (4.3)***	1637 (1.9)***
No insurance	2473 (20.5)	5266 (25.0)***	27,554 (32.0)***
Primary source of support at application			
Personal income (earnings, interest, dividends, rent)	332 (2.7)	638 (3.0)***	5103 (5.9)***
Family and friends	9102 (75.4)	13,929 (66.0)***	67,383 (78.4)***
Public support (SSI, SSDI, TANF, etc.)	2477 (20.5)	6176 (29.3)***	11,605 (13.5)***
All other sources (e.g., private disability insurance and private charities)	162 (1.3)	359 (1.7)***	1881 (2.2)***
Secondary student at the time of application	6295 (52.1)	10,281 (48.7)***	43,086 (50.1)***
Highest level of education at application was high school completion or less	10,827 (89.7)	20,133 (95.4)***	76,885 (89.4)
Advancement of PSE^b^	1451 (13.4)	1498 (7.4)***	16,978 (22.1)***
VR characteristics			
Most significant disability	8546 (70.8)	14,521 (68.8)***	39,600 (46.1)***
Referral source was elementary or secondary school	6360 (52.7)	11,390 (54.0)	47,241 (54.9)***
Mean days between signing of IPE and exit (mean)	11,702 (791.5)	20,649 (887.0)***	85,166 (926.4)***
Receipt of VR services			
Job placement	4792 (41.4)	7384 (37.1)***	25,292 (31.2)***
On-the-job supports for supported employment	2849 (24.7)	4912 (24.6)	5330 (6.6)***
On-the-job supports, short term	2506 (21.9)	3446 (17.5)***	8695 (10.8)***
Job search assistance	4289 (37.3)	6666 (33.5)***	27,754 (34.3)***
Job readiness training	3016 (26.3)	5931 (30.1)***	16,229 (20.2)***
VR counseling	7246 (63.1)	11,533 (58.2)***	53,358 (66.1)***
Education covariate of interest			
PSE training services from VR^c^	2107 (18.3)	3034 (15.3)***	26,028 (32.0)***
Outcome of interest			
Exited with a successful employment outcome	7110 (58.9)	10,730 (50.8)***	46,404 (54.0)***

This table displays the demographic and vocational rehabilitation service use variables for transition age youth (TAY) with autism, TAY with other intellectual and developmental disabilities (IDD), and all other TAY. Significance testing was performed, and asterisks denote the distributions that were significantly different for TAY with IDD and all other TAY compared to TAY with autism

Significance testing was performed comparing TAY with IDD and TAY with all other disabilities to TAY-ASD using logistic regression **p < 0.01; ***p < 0.001

^a^Other IDD includes youth with a cause of disability of intellectual disability, traumatic brain injury, or Cerebral Palsy

^b^Advancement of PSE is defined as the accumulation of any PSE, with or without degree, and only reported for those who reported their highest level of education at application was high school completion or less

^c^PSE training for graduate college, 4-year college, junior college, or vocational school

Because large weights may cause anomalies in analysis, the weights were assessed for any outliers (Austin and Stuart 2015). We examined the range of weights and decided to trim weights based on distribution. Trimmed weights exclude those observations that had a weight below 1% or above 99% of the distribution of weights for the treatment and control groups separately. This method dropped 1.8% of observations, or 211 of the 11,530 TAY-ASD who had information on receipt of PSE training services. Trimming weights allowed for better overlap between propensity scores of the treatment and control groups.

We then used trimmed weighted logistic regression to model the impact of PSE training on exit with employment, while presenting associations with other covariates as well. There is some discussion as to whether covariates should be included in the outcome model when determining the relationship between the treatment (PSE training services) and outcome (exit with employment). Some suggest that including covariates makes the model doubly robust, allowing for more misspecification and the exclusion of unobserved data while giving the best chance for a properly specified model (Bang and Robins 2005; Hullsiek and Louis 2002). Therefore we included additional covariates in the outcome model. All analyses were conducted in Stata 15, propensity score calculation used the *twang* add on for Stata.

## Results

### Research Aim 1: Prevalence of Receipt of PSE Training Services Among TAY-ASD Compared to TAY with Other Disabilities

Table 1 displays the characteristics of TAY. The mean age of TAY-ASD was 19 years at the time of application to services. More TAY-ASD were male (83.1%) and white (85.1%) and fewer Hispanic (6.8%) than TAY with IDD (57.0% male, 65.3% white, 10.4% Hispanic) or all other TAY (57.7% male, 73.0% white, 14.8% Hispanic). Fewer TAY-ASD received SSDI (5.7%) or SSI (29.0%) at application than TAY with IDD (SSDI 7.2%, SSI 40.5%) but more than all other TAY (SSDI 3.5%, SSI 14.4%). TAY-ASD spent less time in VR services between individualized plan for employment (IPE) signature (formal signing of the employment plan preceding services) and case closure (mean = 791.5 days) than TAY with IDD (887.0 days) or all other TAY (926.4 days). About half (52.7%) were referred to VR from secondary or elementary school, and most (70.8%) had a “most significant” disability (compared to 68.8% of TAY with IDD and 46.1% of all other TAY). More TAY-ASD exited VR with employment (58.9%) than TAY with IDD (50.8%) or TAY with all other disabilities (54.0%). About half of TAY-ASD (52.1%), TAY with IDD (48.7%), and all other TAY (50.1%) were secondary students at the time of VR application. Most of TAY-ASD (89.7%), TAY with IDD (95.4%), and all other TAY (89.4%) had high school completion or less as their highest level of education. About one-fifth (18.3%) of TAY-ASD received some PSE training, compared to 15.3% of TAY with IDD and 32.0% of all other TAY. Of those who had high school as their highest level of education, fewer TAY-ASD (13.4%) advanced PSE experiences during VR than all other TAY (22.1%), but more than TAY with IDD (7.4%).

### Research Aim 2: Characteristics of PSE Training Service Recipients

TAY-ASD who received PSE training services were younger, less likely to be receiving SSI at application, less often had a most significant disability, and less often received on-the-job supports for supported employment or short term on-the-job supports than TAY-ASD who did not receive PSE training. They were more often referred by an elementary or secondary school, spent more days in VR between IPE signature and exit, and more often received VR counseling services (Table 2).

**Table 2:** Unadjusted comparison of transition age youth with autism who received or did not receive PSE training services

	TAY-ASD who received PSE training (n = 2107)	TAY-ASD who did not receive PSE training (n = 9423)
	n (%)	n (%)
Demographic characteristics		
Age (mean)	2107 (18.4)	9423 (19.1)***
Gender, male	1724 (81.8)	7855 (83.4)
Race		
White	1843 (87.5)	7982 (84.7)
Black	160 (7.6)	955 (10.1)
Other/multiple	104 (4.9)	486 (5.2)
Ethnicity—Hispanic or Latino	123 (5.8)	641 (6.8)
Received SSDI at application	87 (4.1)	553 (5.9)**
Received SSI at application	422 (20.0)	2878 (30.5)***
Type of insurance at time of application		
Private	999 (47.4)	4071 (43.2)
Public	518 (24.6)	3059 (32.5)
Both private and public	103 (4.9)	538 (5.7)
No insurance	487 (23.1)	1755 (18.6)
Primary source of support at application		
Personal income (earnings, interest, dividends, rent)	78 (3.7)	247 (2.6)***
Family and friends	1700 (80.7)	7032 (74.6)***
Public support (SSI, SSDI, TANF, etc.)	309 (14.7)	2020 (21.4)***
All other sources (e.g., private disability insurance and private charities)	20 (0.9)	124 (1.3)***
Secondary student at the time of application	1144 (54.3)	4851 (51.5)
Highest level of education at application was high school completion or less	1883 (89.4)	8430 (89.5)
VR characteristics		
Most significant disability	1354 (64.3)	6749 (71.6)***
Referral source was elementary or secondary school	1271 (60.3)	4777 (50.7)***
Mean days between signing of IPE and exit (mean)	2106 (1259.1)	9089 (673.5)***
Receipt of VR services		
Job placement	813 (39.7)	3889 (41.3)
On-the-job supports for supported employment	296 (14.5)	2460 (26.1)***
On-the-job supports, short term	384 (18.9)	2116 (22.5)***
Job search assistance	779 (38.0)	3469 (36.8)
Job readiness training	481 (23.6)	2532 (26.9)**
VR counseling	1361 (66.8)	5857 (62.2)***
Outcome of interest		
Exited with a successful employment outcome	1336 (63.4)	5531 (58.7)***

In the table compares demographic and vocational rehabilitation service variables in transition age youth with autism (TAY-ASD) who did or did not receive PSE training services. Statistical comparisons are presented and denoted with asterisks

Significance testing was performed comparing TAY-ASD who did receive PSE training and TAY-ASD who did not using logistic regression **p < 0.01; ***p < 0.001

### Research Aim 3: Association of PSE Training Services with Employment

In unadjusted comparisons (Table 2), TAY-ASD who received PSE training services from VR more often exited VR with employment than TAY who did not. The propensity score weighted logistic regression model revealed that the odds of exiting VR with employment was 1.59 times higher for TAY-ASD who received PSE training versus those who did not (OR 1.59; 95% CI 1.38, 1.84; *p* < 0.001).

Use of GBM to estimate propensity scores resulted in balance of these groups (Table 3) after weighing in all but one covariate (receipt of SSI payments at application). Standardized effect sizes were very small for all covariates after matching, with all values less than 0.1 SD difference between the means of the two groups indicating that the groups were well matched after propensity score weighting.

**Table 3:** Balance statistics for transition age youth with autism who received PSE training services and those who did not after weighting on propensity score

Variable	TAY-ASD who received PSE training services		TAY-ASD who did *not receive* PSE training services		Standard effect size^a^	Kolmogorov–Smirnov (KS) test statistic	*p* Value
	Mean	SD	Mean	SD			
Age	18.877	2.109	18.994	2.174	− 0.055	− 1.525	0.127
Gender	1.167	0.373	1.168	0.374	− 0.002	− 0.068	0.946
Race	1.184	0.490	1.199	0.511	− 0.030	− 0.969	0.332
Hispanic ethnicity	0.056	0.231	0.066	0.248	− 0.040	− 1.401	0.161
SSI at application	0.251	0.434	0.287	0.453	− 0.081	− 2.457	0.014
Type of insurance	1.968	1.127	1.995	1.123	− 0.024	− 0.800	0.424
Primary source of support at application	2.180	0.464	2.199	0.489	− 0.041	− 1.257	0.209
Most significant disability	0.716	0.451	0.703	0.457	0.027	0.923	0.356
Referral source was secondary education institution	0.544	0.498	0.524	0.499	0.040	1.216	0.224
Above the mean number of days from IPE signature to closure	0.398	0.490	0.374	0.484	0.050	1.746	0.081
Receipt of job placement services	0.404	0.491	0.409	0.492	− 0.011	− 0.340	0.734
Receipt of on-the-job supports for SE	0.221	0.415	0.240	0.427	− 0.045	− 1.210	0.226
Receipt of short term on-the-job supports	0.203	0.402	0.218	0.413	− 0.037	− 1.171	0.242
Receipt of job search services	0.371	0.483	0.369	0.482	0.004	0.137	0.891
Receipt of job readiness training services	0.279	0.449	0.263	0.440	0.037	1.026	0.305
Receipt of VR counseling services	0.652	0.476	0.629	0.483	0.048	1.478	0.139
Secondary student at time of application	0.545	0.498	0.523	0.499	0.044	1.355	0.175

In the table displays the balance statistics of the propensity score weight calculations. Balance was assessed between the treatment (received PSE training) and control (did not receive PSE training) groups on a variable by variable basis. The mean and standard deviation for each variable is presented by group, as well as the standardized effect size and the Kolmogorov–Smirnov test statistic and its corresponding *p* value. All variables were balanced between groups after weighting except receipt of SSI at application

The balance was assessed on a variable by variable basis. These tables show the extent to which the model succeeded in adjusting both the control and treatment groups so that their weighted characteristics balance with one another

^a^Standardized effect size was calculated as the treatment group mean minus the control group mean divided by the pooled sample (treatment and control) standard deviation

The mean of the weights for the group who received PSE training was 4.84 (SD, 4.85) with a range of 1–105.16. For the group who did not receive PSE training the mean of the weights was 1.21 (0.25) with a range of 1.001–3.63. Trimming reduced the largest weight to 22.66 for TAY-ASD who received PSE training and 2.13 for TAY-ASD who did not receive PSE training.

## Discussion

This study examined the importance of VR in supporting PSE among TAY-ASD. Eighteen percent of TAY-ASD received PSE supports—less than TAY with other disabilities (32%) but more than TAY with IDD (15%). This finding is consistent with previous research that TAY with IDDs or psychiatric disabilities are less likely to receive PSE training services than TAY with physical disabilities, but TAY with IDDs see the strongest jump in attaining employment when these services are received, suggesting PSE training services should be better utilized in this group (O’Neill et al. 2015). Low rates of PSE in TAY-ASD are documented (Newman et al. 2011; Roux et al. 2015), but it is increasingly understood that PSE is valuable for meaningful and stable employment.

TAY-ASD who received PSE training services from VR were younger, with less significant job impediments, and were less likely to be receiving VR services that are typically associated with better employment outcomes. Even though use of PSE training services through VR was low in TAY-ASD, it was positively associated with employment; the odds of exiting VR with employment were more than 1.5 times greater for TAY-ASD who received PSE training than those who did not. Previous research has shown that receipt of PSE training services and participation in PSE are also associated with higher hourly earnings for TAY-ASD (Migliore et al. 2012).

The results of this study establish that TAY-ASD who receive PSE training services through VR are more likely to exit with employment and highlights the underutilization of PSE for TAY-ASD compared to other TAY who use VR services. These are important steps in increasing PSE attendance in TAY-ASD. However, we cannot describe the earnings or longevity of this employment.

The low utilization of PSE training in TAY-ASD is an important avenue for future inquiry. There are several potential explanations. In general, people who pursue PSE may not know that VR provides educational supports. So youth who are seeking PSE may not apply for VR services. Furthermore, VR agencies may not provide information about PSE training as an option for youth, particularly those who are not on a typical PSE trajectory. This could explain the low incidence of PSE training in TAY-ASD and TAY with IDD, particularly compared to TAY with other disabilities. The lower receipt of SSI and the smaller proportion of most significant disability status in TAY-ASD who received PSE training may suggest lower severity of impairment in TAY-ASD who received PSE training than for TAY-ASD who did not. Although PSE may not be ideal for all TAY-ASD (Shattuck et al. 2012a), with proper supports and accommodations it may be a good option for many and may increase access to better jobs. Given the lack of available public services for supporting PSE, VR may be a key to increasing advancement of PSE for TAY-ASD.

Further research is needed to understand whether PSE training is associated with longevity of employment or associated earnings. It is possible that VR could play the role of connecting more TAY-ASD to PSE opportunities as a means of maximizing employment outcomes. Research into this would need to include identifying the kind of supports and accommodations that would aid in increasing the proportion of TAY-ASD that receive PSE training. Barriers to implementing PSE training services may lie in the disconnection between VR providers and PSE institutions, a resource and knowledge gap that could prevent effective collaboration (Plotner and Marshall 2016). This and other barriers must be addressed in future research and practice.

While this study focuses on the role of PSE in supporting employment, there are several other possible explanations for low employment in TAY-ASD. These include poor transition planning for life after high school, limited interagency coordination, and lack of understanding of ASD by VR and other agencies and service providers (Kuo et al. 2018; Roux et al. 2018a).

This study had several limitations. First, the variables available in the RSA-911 data set to capture disability characteristics and employment outcomes are limited. Measurement of severity of impairment, for example, is limited to the notation of a “most significant disability.” This limits our ability to factor severity into propensity score weights. Receipt of SSI, another potential indicator of severity of impairment, did not balance after propensity score methods, noting that the groups still differed significantly on this variable after weighting. Employment status is also simply measured as the presence or absence of employment at VR exit, and we do not have information about wages or employment longevity. Further, we do not have information about household income or formal and informal networks, which are related to employment success. While their rate of PSE training services is low, TAY-ASD are more likely to exit VR with employment than their peers. Some of these unmeasured variables may be responsible for this finding. Second, we are limited in our understanding of what each state provides in terms of PSE training services, how they are provided, and to what extent. Third, VR data is administrative. While the rates of missingness were low, the nature of administrative entry leads to unknown accuracy and reliability of personal information. Finally, RSA-911 data contains information about applicants and clients of VR, so it is not representative of all TAY-ASD, only those who use VR services. The findings are therefore not inferential to the general population.

This study also has several strengths. It is among the first to connect employment of TAY-ASD with receipt of PSE services. This is important for establishing expectations for PSE attainment in TAY-ASD. Second, the propensity score methods used in this study aim to control for individual characteristics and make a causal linkage between PSE training and exit with employment. Third, the comparison to TAY with IDD and TAY with all other disabilities provides context as to how TAY are using VR PSE training services.

## Conclusion

This study provides evidence of an association between PSE and employment for TAY-ASD who use VR services. Even though PSE training services are positively associated with successful employment, the receipt of PSE training services is low in TAY-ASD who receive VR services. Establishing the importance of VR as a connector to PSE in TAY with disabilities is important to increasing access to PSE in this population. Acknowledging the importance of PSE in job acquisition, including for TAY with disabilities, is integral to employment success. Future research could examine the nature of PSE training services in VR and how different service provision impacts employment success.
